# The Elimination and Carcinogenic Potency of 3:4-Benzyprene in Mice after Subcutaneous Injection of Non-Lipoid Solutions

**DOI:** 10.1038/bjc.1947.42

**Published:** 1947-12

**Authors:** H. Weil-Malherbe


### THE ELIMINATION AND CARCINOGENIC POTENCY OF 3:4-BENZPYRENE IN MICE AFTER SUBCUTANEOUS INJECTION OF NON-LIPOID SOLUTIONS.

H. WEIL-MALHERBE.*

*From the Department of Physiology, Medical School, King's College, Newcastle-upon-Tyne.*

Received for publication November 27, 1947.

THE introduction of 1:3:7:9-tetramethyluric acid as a solubilizer for 3:4-benzpyrene (Weil-Malherbe, 1946) has made it possible to prepare aqueous solutions of benzpyrene suitable for injection. If solubilizer solutions of equal concentration (weight/volume) are compared, a tetramethyluric acid solution will dissolve about five times more benzpyrene than a caffeine solution and about 10 times more than a deoxycholate solution.

It has been suggested that the solubilization of hydrocarbons by purines is connected with the formation of coordination complexes (Weil-Malherbe, 1946). A similar mechanism was assumed to underlie the activation of elimination and carcinogenicity observed when benzpyrene is injected in solvents containing cholesterol or cholestanol (Weil-Malherbe, 1947). It was therefore of interest to study the rate of elimination and the carcinogenic activity of solubilized benzpyrene. It was anticipated that in the absence of a stable lipoid depot the dispersion of benzpyrene and its absorption by the surrounding tissues would be greatly speeded up. The additional effect of the solubilizer can only be gauged if allowance is made for the different physical conditions resulting from the absence of a lipoid depot. The rate of elimination after the injection of colloidal aqueous or ethereal solutions of benzpyrene was therefore also measured. Peacock and Beck (1938) obtained only a few tumours with 0·5–1 mg. benzpyrene subcutaneously administered to mice either as the powdered solid or in ethyl ether solution, and they attributed this to a more rapid dispersal and elimination of the hydrocarbon. The effects of aqueous solutions of benzpyrene made up with the aid of a solubilizer do not appear to have been adequately studied. Gummel and Rarei (1939) used sodium cholestenone sulphonate solutions as a solvent for benzpyrene, but their method of preparation (solution of benzpyrene in acetone, addition to cholestenone sulphonate solution and evaporation of acetone) suggests that benzpyrene may have been present partly in colloidal solution. They found after five months, as a result of daily injections in rabbits, chronic degenerative changes in parenchymatous tissues, such as amyloidosis of spleen and liver, necrosis of liver cells and testicular atrophy.

### EXPERIMENTAL.

1:3:7:9-Tetramethyluric acid was prepared according to Biltz and Strufe (1916).

Tetramethyluric acid solutions of benzpyrene were prepared by shaking the

* Present address : Runwell Hospital, Wickford, Essex.

purine solution with an excess of solid hydrocarbon for 3–4 hours at room temperature. If the concentration of tetramethyluric acid was higher than 3 per cent the mixtures were warmed on a water bath to about 50° C. and kept there for half an hour with constant stirring. Before use the solutions were filtered through Whatman No. 5 filter paper. Solutions containing 4 or 5 per cent tetramethyluric acid were kept at body temperature to prevent crystallization.

Deoxycholate solutions of benzpyrene were prepared in a similar way.

A colloidal solution of benzpyrene was prepared by the method of Feigenbaum (1944). It was filtered before use.

Benzpyrene was estimated fluorimetrically (Weil-Malherbe, 1944).

Glaxo FF mice of both sexes were used for the experiments.

### *Elimination Rates.*

It was considered desirable to introduce as little variation of technique as possible. The same volume of solution as that used in the experiments with lipoid solvents, 0·3 ml., was therefore adhered to for the injections. A 5 per cent solution of tetramethyluric acid which, at body temperature, is practically saturated will dissolve about 0·2 mg. of benzpyrene in 0·3 ml., while 0·3 ml. of a 4 per cent solution contain about 0·15 mg. of benzpyrene. The 5 per cent solution, if injected in amounts of 0·3 ml., is appreciably toxic, causing a mortality of about 30 per cent, whereas the mortality is only about 7 per cent with the 4 per cent solution. The latter was therefore chosen for the elimination experiments.

A solution of sodium deoxycholate of the same concentration (4 per cent) only dissolves about 15 $\mu$g. benzpyrene per 0·3 ml. and, moreover, causes skin damage (haemorrhagic oedema followed by necrosis) in a number of cases. The subcutaneous injection of 0·3 ml. ether solution also proved rather toxic, and led to deep anaesthesia of all and the subsequent death of 21 out of 35 mice.

In all four series cases of skin necrosis were observed which developed near the site of injection after 2–4 weeks. The necrotic patch of skin usually showed a brilliant bluish-white fluorescence in ultraviolet light. A scab usually developed which was finally sloughed off. The fluorescent substance was lost in the process, and analyses showed that there was practically no benzpyrene left in such mice. Because of the prevalence of this phenomenon, especially in the ether series, the analyses had to be confined to the first 4–5 weeks. In the tetramethyluric acid series the majority of estimations performed after the eighth day showed that the benzpyrene content had fallen below the level of sensitivity of the method. To avoid an undesirable bias the estimations which gave a measurable result after this date were omitted from the statistical analysis, but they are shown in Fig. 1. In Fig. 1–4 the values of log $S$ (= $\mu$g. benzpyrene found) were plotted against the time $t$ (days after injection). The straight line represents the linear regression of log $S$ on $t$, calculated by the method of least squares. The regression coefficients and their standard errors are given in Table I.

It is obvious that the rate of elimination in the two experiments in which solubilizers were used is of quite a different order from that observed in the other two experiments. The differences within these two groups are not

FIG. 1.—Elimination rate of 3:4-benzpyrene after subcutaneous injection of a saturated solution in 4 per cent aqueous tetramethyluric acid solution. Only the values to the left of the broken line were used for the calculation of the linear regression. Bracketed points represent analyses in which no measurable amounts of benzpyrene could be detected.

FIG. 2.—Elimination rate of 3:4-benzpyrene after subcutaneous injection of a saturated solution in 4 per cent aqueous sodium deoxycholate solution.

statistically significant ($P > 0{\cdot}05$). With ethereal or colloidal solutions the elimination proceeds at practically the same rate as was observed with various lipoid solvents, at least within the first 4–5 weeks. After this time ulceration may intervene and cause a sudden and almost complete loss of benzpyrene.

FIG. 3.—Elimination rate of 3:4-benzpyrene after subcutaneous injection of a colloidal solution.

FIG. 4.—Elimination rate of 3:4-benzpyrene after subcutaneous injection of a solution in ethyl ether.

TABLE I.—*Elimination of 3 : 4-Benzpyrene Dissolved in Non-lipoid Vehicles After Subcutaneous Injection.*

| Solution. | Initial amount of benzpyrene. (μg. in 0·3 ml.) | Number of observations. | Linear regression coefficient. | Standard error. |
|---|---|---|---|---|
| 4% tetramethyluric acid | 160 | 25 | — 0·214 | 0·0532 |
| 4% sodium deoxycholate | 14·3 | 16 | — 0·127 | 0·0234 |
| Colloidal | 100 | 20 | — 0·0388 | 0·00677 |
| Ethyl ether | 307 | 13 | — 0·0264 | 0·00854 |

This may account for the impression expressed by Peacock and Beck (1938) that absorption and elimination was faster after injection of ether solutions of benzpyrene than it was after injection of lipoid solutions. On the other hand, there is a remarkable increase of the elimination rate in the presence of solubilizers, amounting to an acceleration of about 5–10 times. In Fig. 5 the elimination rates of benzpyrene found in a number of experiments with lipoid and non-lipoid solvents are compared. The elimination constant of — 0·214 for tetramethyluric acid solution corresponds to a half-lifetime of 1·4 days, whereas the constant for tricaprylin, — 0·0157, corresponds to a half-lifetime of 19·1 days.

There is a rather wide scatter which becomes more marked as the elimination rate increases. In the tetramethyluric-acid series an initial divergence from linearity has to be assumed, indicating a rapid drop in the first few hours after injection to a level from which the further elimination proceeds in a linear fashion. Fairly high values of benzpyrene persist in a few sporadic cases even after the

FIG. 5.—Elimination rates of 3:4-benzpyrene dissolved in different solvents after subcutaneous injection :
(1) From this paper.
(2) From Weil-Malherbe (1947).
(3) From Weil-Malherbe & Dickens (1946).

eighth day. These were cases showing necrotic skin areas impregnated with fluorescent material. Any benzpyrene trapped in the necrotic tissues presumably remains fixed there. If this interpretation is correct, the analyses of such cases are immaterial to the evaluation of the elimination constant and may be neglected.

*Metabolism of Benzpyrene after Injection in Tetramethyluric Acid Solution.*

In view of the increased elimination rate of benzpyrene after its injection in tetramethyluric acid solution the question arose whether a change in its metabolism took place. In particular the possibility of a urinary excretion of a complex between tetramethyluric acid and either unchanged or oxidized benzpyrene had to be considered. Twenty rats were therefore injected daily with

1 ml. of a 4 per cent solution of tetramethyluric acid saturated with benzpyrene for 12 days. An estimated total of 100 mg. benzpyrene was thus administered. The rats were kept in two metabolism cages, and their urine was collected in brown bottles containing 20 ml. 10 N sulphuric acid. The combined urine was filtered and extracted ten times with ether in a separating funnel. The pooled ether extracts were washed with water, dried and evaporated. After extraction the urine was neutralized with baryta, filtered and concentrated *in vacuo* to 300 ml. It was then extracted with chloroform in a separating funnel.

The residue from the ether extract was taken up in benzene, and the benzene solution extracted with a small volume of N sodium hydroxide solution. The soda extract was saturated with solid ammonium sulphate and re-extracted with benzene (phenolic fraction). Both benzene solutions, the phenolic and the neutral fraction, were dried and passed through columns of silica gel. The filtrates were then passed through columns of alumina and developed with benzene. No trace of unchanged benzpyrene or of benzpyrene quinones was detected in the neutral fraction. The phenolic fraction, after filtration through silica gel, showed a bluish-white fluorescence ; on the alumina column a band with yellow fluorescence formed and moved slowly down. This was taken as an indication of the presence of a phenolic metabolite, but the substance was present in traces only and could not be further characterized.

The benzene insoluble residue of the ether extract slowly crystallized. On further investigation, which need not be described in detail, it appeared mainly to consist of a mixture of methylmalonic (Boyland and Levi, 1936) and succinic acids.

Several crystalline fractions were also isolated from the chloroform extracts. Most of them were nitrogen-free and were not identified. Small quantities of two nitrogen-containing substances (m.p. 157 and 206° C.) were not identical with tetramethyluric acid.

The result of the metabolism experiment is in agreement with the prevailing views on the fate of injected benzpyrene, the bulk of which is apparently excreted with the faeces in the form of a phenolic derivative, and no unchanged benzpyrene and only traces of the phenolic metabolite pass into the urine (Berenblum and Schoental, 1943). The situation, at least as far as urinary excretion is concerned, does not seem to be modified by the presence of tetramethyluric acid. It is likely, therefore, that the increase of the elimination rate is largely a local effect, perhaps due to a more rapid penetration across the cell membranes. It is interesting to note that no unchanged tetramethyluric acid was found in the urine either. As is well known, methyluric acids may undergo partial or complete demethylation at one or several positions of the molecule within the body (Myers and Hanzal, 1946).

### *Carcinogenic Activity of* 3:4-*Benzpyrene Dissolved in Aqueous Tetramethyluric Acid Solution.*

A series of mice were given a single injection of 0·3 ml. of a 5 per cent solution of tetramethyluric acid in water, saturated with benzpyrene. The amount of benzpyrene injected in this way was about 0·2 mg. per mouse. Among 21 mice alive after 8 months only one sarcoma arose locally, and this appeared 5 months after the injection.

In a second experiment a series of 20 mice were injected subcutaneously with 0·3 ml. of a 1 per cent aqueous solution of tetramethyluric acid saturated with benzpyrene, while an equal amount of a 1 per cent tetramethyluric acid solution free from benzpyrene was injected into another series of 20 mice. The injections were repeated twice weekly for 32 weeks. As a single dose contained about 10 $\mu$g. benzpyrene, each mouse received altogether about 0·32 mg. The experiment, which is now in its seventh month, has produced one local tumour so far.

### DISCUSSION.

The results show two points of interest : (1) When benzpyrene is injected in colloidal or ethereal solution, its elimination proceeds at a rate not much different from that observed with several oily solutions (Fig. 5). The dispersal and absorption of benzpyrene do not therefore seem to be greatly facilitated by this form of application or, if they are, do not thereby appreciably accelerate the rate of elimination. Little is known about the carcinogenic response to these solutions. Morton and Mider (1939) observed in C 57 black mice an incidence of 50 per cent tumours after the subcutaneous injection of 0·25 mg. benzpyrene in colloidal solution, as compared with an incidence of 78 per cent after the injection of a sesame oil solution. It may be recalled that sesame oil has a procarcinogenic effect (Dickens and Weil-Malherbe, 1946) ; the incidence observed with the colloidal solution is close to the tricaprylin norm. Peacock and Beck (1938) found no tumours six months after the subcutaneous injection of mice with 0·5 mg. benzpyrene in 0·1 ml. ether. This is a surprising result. In the writer's experiments the injection of ethereal solutions led to frequent skin necrosis and demarcation, but no mention of it is made by Peacock and Beck. A reinvestigation with a careful watch for leakage or ulceration seems desirable.

(2) The elimination of benzpyrene is considerably faster when it is injected in an aqueous solution of a solubilizer than in a colloidal or ethereal solution. This result is unexpected for the following reasons : (1) The solubilization of benzpyrene increases with the square of the concentration of tetramethyluric acid (Weil-Malherbe, 1946) or sodium deoxycholate (Weil-Malherbe, unpublished) in the concentration range used. Precipitation is therefore caused by dilution, such as takes place when the injected solution mixes with the tissue fluids. (2) Addition of electrolytes also leads to precipitation. (3) The complexes between solubilizer molecules and benzpyrene molecules are assumed to be largely dissociated in solution (Weil-Malherbe, 1946), and a more rapid diffusion of the water-soluble solubilizer molecule in the tissues might be expected, a process which would also lead to precipitation of benzpyrene.

One would anticipate, therefore, that benzpyrene would be deposited and subsequently taken up in combination with, or solution by, tissue components in a similar manner, whether dissolved in ether, in colloidal solution or in an aqueous solution of a solubilizer. The difference in the rate of elimination, however, leads to the conclusion that this is not the case, and that the interaction between solubilizer and hydrocarbon persists to some extent after injection, presumably long enough in some way to assist the transport of the hydrocarbon across the cell membranes.

In the case of cholesterol and cholestanol a correlation has also been postulated between the increased rate of elimination of benzpyrene and the formation of association complexes (Weil-Malherbe, 1947). But, whereas here the speedier elimination was associated with a higher tumour incidence, the tumour incidence is negligible with tetramethyluric acid solutions of benzpyrene. A number of possible explanations offer themselves: (1) There may be a rate of metabolism of benzpyrene which is optimal for carcinogenesis, and this optimum may be exceeded in the case of tetramethyluric acid solutions. If this is so, not only the intensity, but also the duration of the process would be of importance. It need not be implied in this assumption that the metabolic reactions are a necessary cause of the carcinogenic stimulus; they may merely be a corollary to a simultaneous but unrelated activation of the reactions which initiate the carcinogenic metamorphosis. (2) The total quantities of benzpyrene injected or the effective concentrations in the tissue may have been insufficient. The dose used in the single-injection experiment was not a great deal below that which is found to be effective when applied in an oily vehicle. But a large proportion of it was eliminated during the first few hours after the injection. This sudden drop in benzpyrene content suggests that part of the injected fluid may have escaped into the circulation, and was therefore metabolized and eliminated at a much faster rate than the benzpyrene which remained in the subcutaneous tissues. In the mice which were given repeated injections of small doses of benzpyrene the chances of a sufficient local concentration being reached at the site of injection were of course smaller still. This was intentional, as it was the object of the experiment to induce not so much local as remote changes. However, the experiment will be repeated with higher doses.

Shear (1941) obtained only one local sarcoma out of 24 mice of Strains A and D, 16 months after injection of 0·5 ml. of a 0·25 per cent. aqueous solution of the sodium salt of the molecular compound formed by the combination of one molecule of 1:2:5:6-dibenzanthracene with four molecules of deoxycholic acid. A number of multiple lung tumours were observed in Strain A mice. The dose injected, 0·17–0·18 mg. dibenzanthracene, is effective if administered in an oily solvent. In similar experiments with an aqueous solution of the sodium salt of the 20-methylcholanthrene-deoxycholic acid compound, in which 0·5 ml. of a 0·25 per cent solution were injected (about 0·18 mg. methylcholanthrene per injection), two tumours arose amongst six Strain A mice and none in six Strain D mice after three months (Shear, 1936). At that time, and again after four months, the mice were reinjected with the same amount. After six months all Strain A mice and three Strain D mice had developed local tumours. This suggests that, provided the quantity is high enough in relation to the activity, the solubilization of carcinogenic hydrocarbons does not preclude their activity. (3) The solubilization of benzpyrene may bring about a qualitative change of its metabolism. Although the analysis of the urine of rats which had been injected with a benzpyrene-tetramethyluric acid solution gave no indication of a urinary excretion of unchanged benzpyrene or of substantial amounts of a metabolite, the rapid initial drop of benzpyrene content after injection is a digression from the usual course of events, whether the change concerns only the site or also the mechanism of the metabolic transformations.

It is at present impossible to decide which of these explanations is correct. But the three possibilities are by no means exclusive to each other and may all apply together in varying degrees, with the second factor probably predominating.

SUMMARY.

Mice were subcutaneously injected with the following solutions of 3:4-benzpyrene : aqueous solutions made up with the aid of 1:3:7:9-tetramethyluric acid or sodium deoxycholate as solubilizers, an aqueous colloidal solution, and a solution in ethyl ether, and the rate of elimination of benzpyrene was measured. It was found that with the ethereal and colloidal solutions the rate of elimination was of the same order of magnitude as that observed with several lipoid solvents, while it was 5–10 times higher in the experiments in which solubilizers were used. The increase in the elimination rate brought about by tetramethyluric acid is not accompanied by a urinary excretion of unchanged benzpyrene or of substantial amounts of a metabolite, as shown by experiments on rats. No unchanged tetramethyluric acid was detected in the urine either.

Out of 21 mice injected with a single dose of a solution of tetramethyluric acid containing about 0·2 mg. benzpyrene per dose, only one developed a sarcoma after 5 months. One tumour arose in a series of 20 mice injected twice weekly for 32 weeks with a solution of tetramethyluric acid containing 10 μg. benzpyrene per injection.

The results have been discussed.

REFERENCES.

Berenblum, I., and Schoental, R.—(1943) *Cancer Res.*, **3**, 145.
Biltz, H., and Strufe, K.—(1916) *Liebigs Ann.*, **413**, 197.
Boyland, E., and Levi, A. A.—(1936) *Biochem. J.*, **30**, 2007.
Dickens, F., and Weil-Malherbe, H.—(1946) *Cancer Res.*, **6**, 161.
Feigenbaum, J.—(1944) *Exp. Med. Surg.*, **2**, 304.
Gummel, H., and Rarei, B.—(1939) *Z. Krebsforsch.*, **48**, 347.
Morton, J. J., and Mider, G. B.—(1939) *Proc. Soc. exp. Biol. N.Y.*, **41**, 357.
Myers, V. C., and Hanzal, R. F.—(1946) *J. biol. Chem.*, **162**, 309.
Peacock, P. R., and Beck, S.—(1938) *Brit. J. exp. Path.*, **19**, 315.
Shear, M. J.—(1936) *Amer. J. Cancer*, **26**, 322.—(1941) Quoted in Hartwell, J. L., 'Survey of Compounds which have been Tested for Carcinogenic Activity.' Bethesda : Federal Security Agency, United States Public Health Service.
Weil-Malherbe, H.—(1944) *Biochem. J.*, **38**, 135.—(1946) *Ibid.*, **40**, 351.—(1947) *Brit. J. Cancer*, **1**.
*Idem* and Dickens, F.—(1946) *Cancer Res.*, **6**, 171.


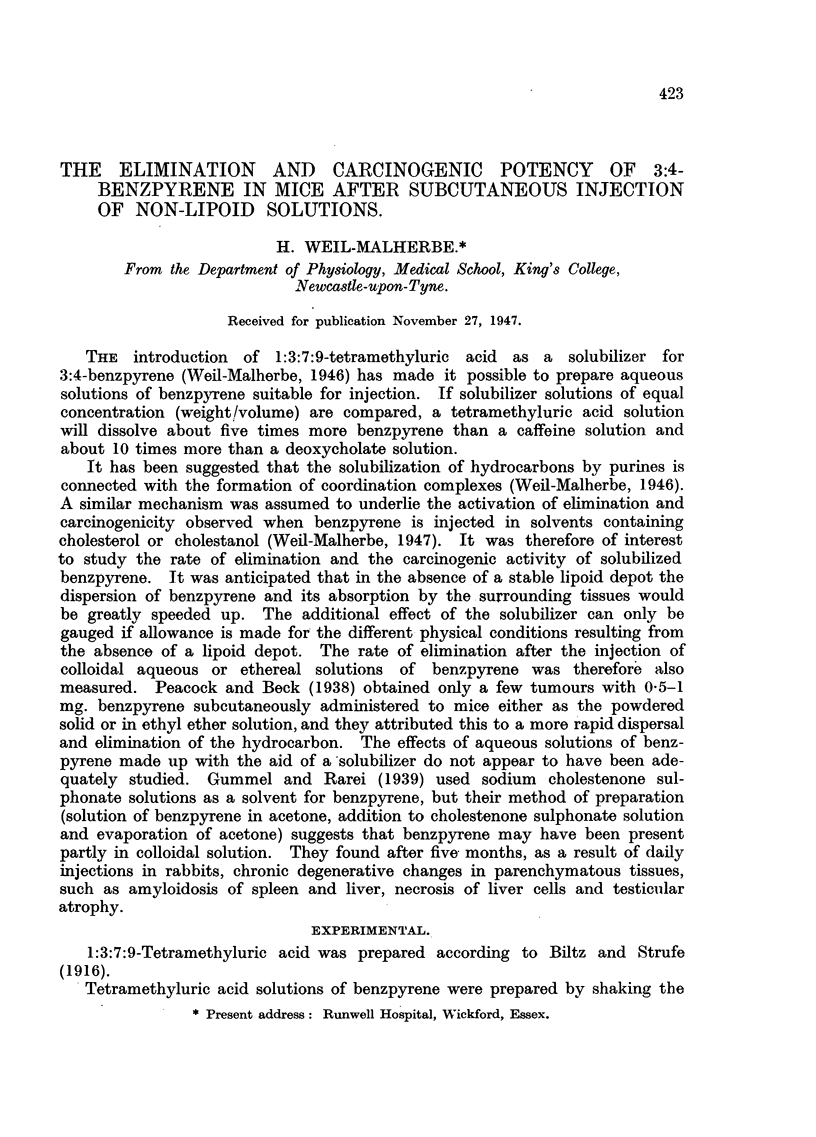

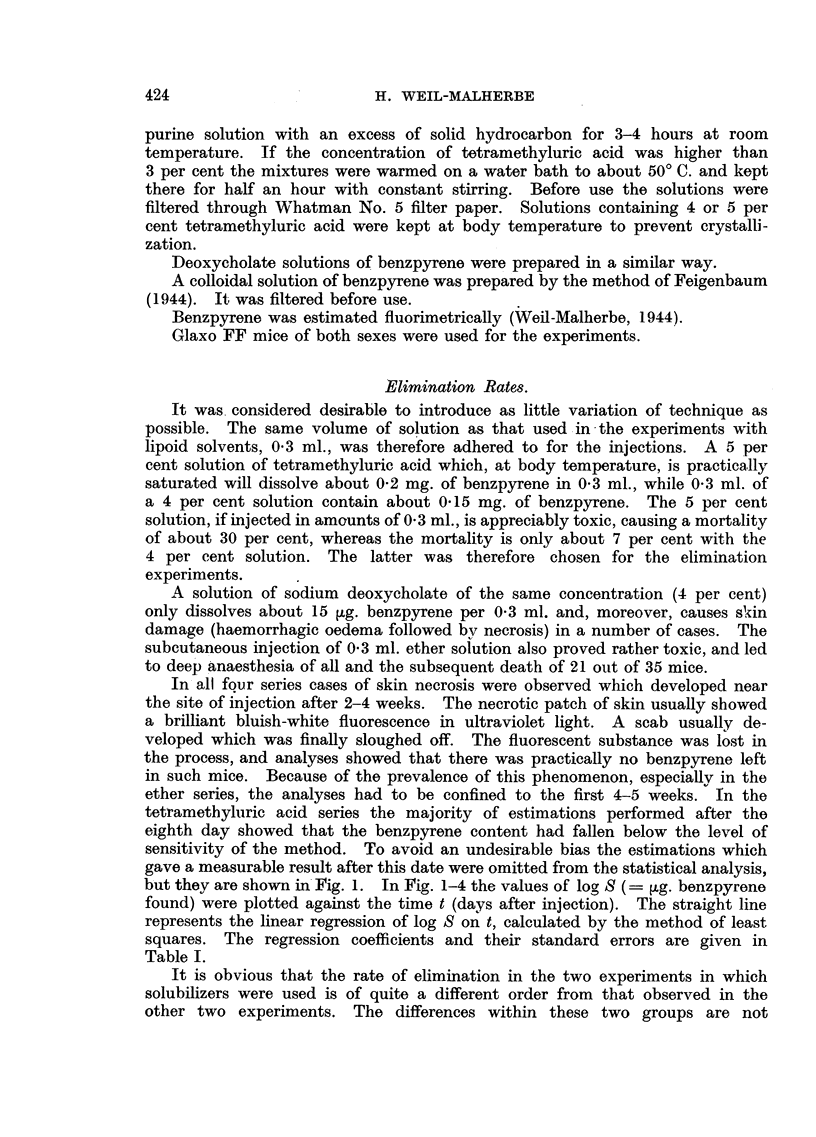

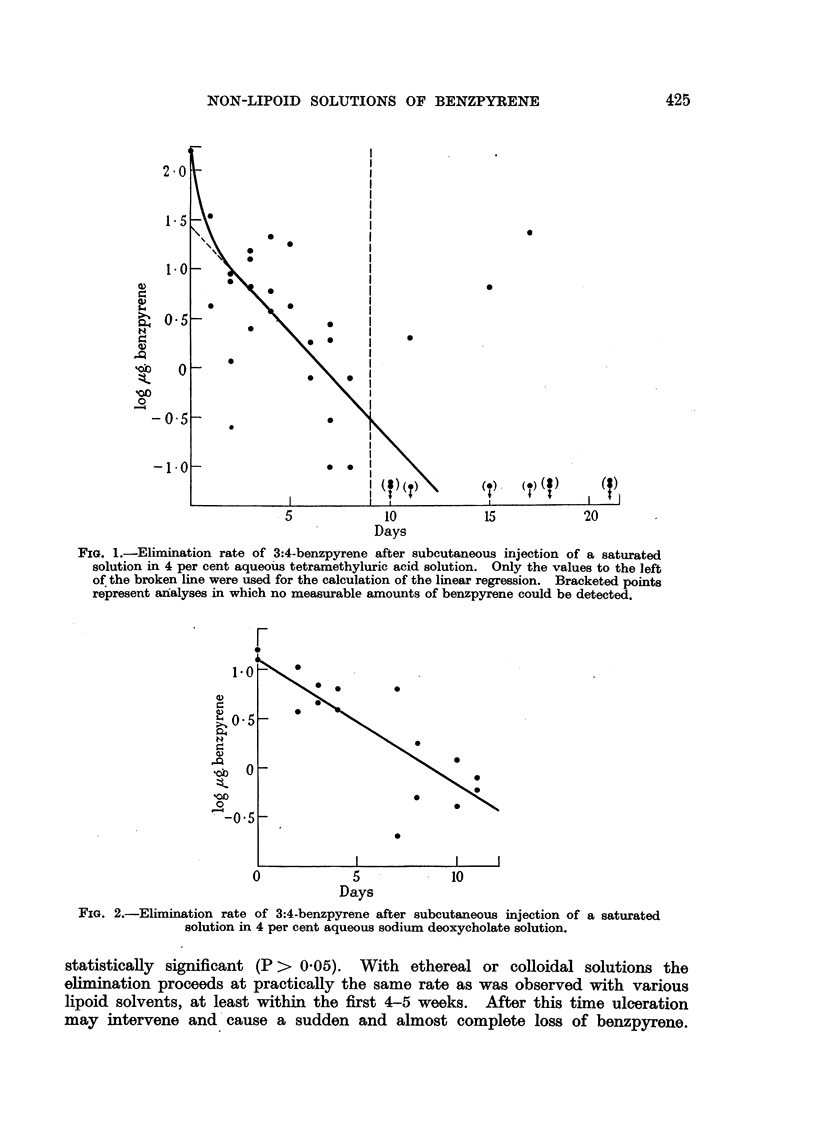

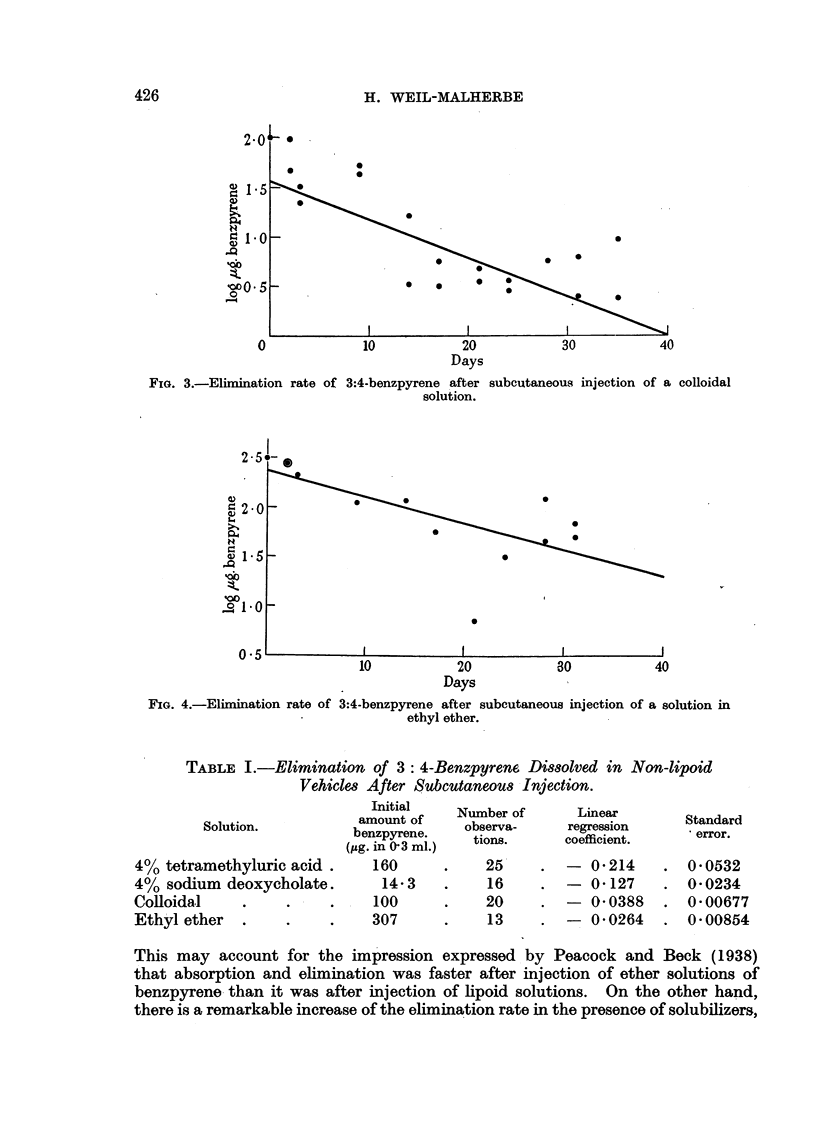

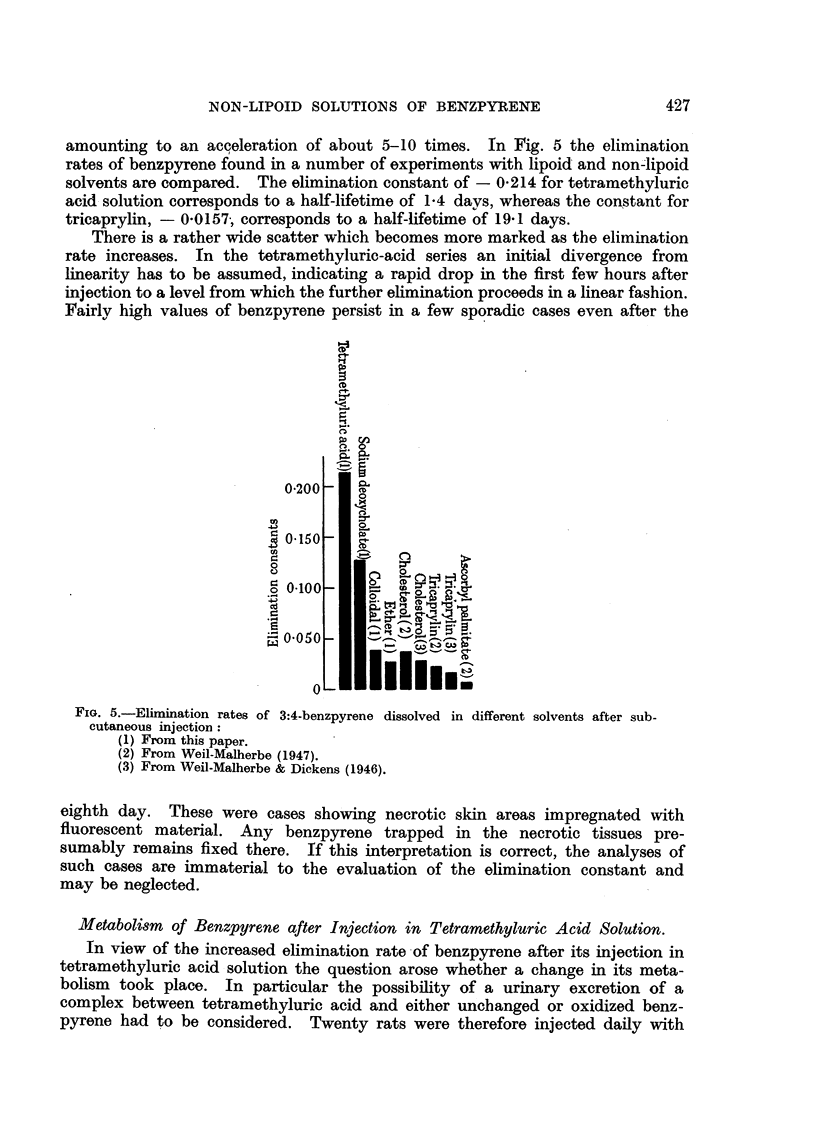

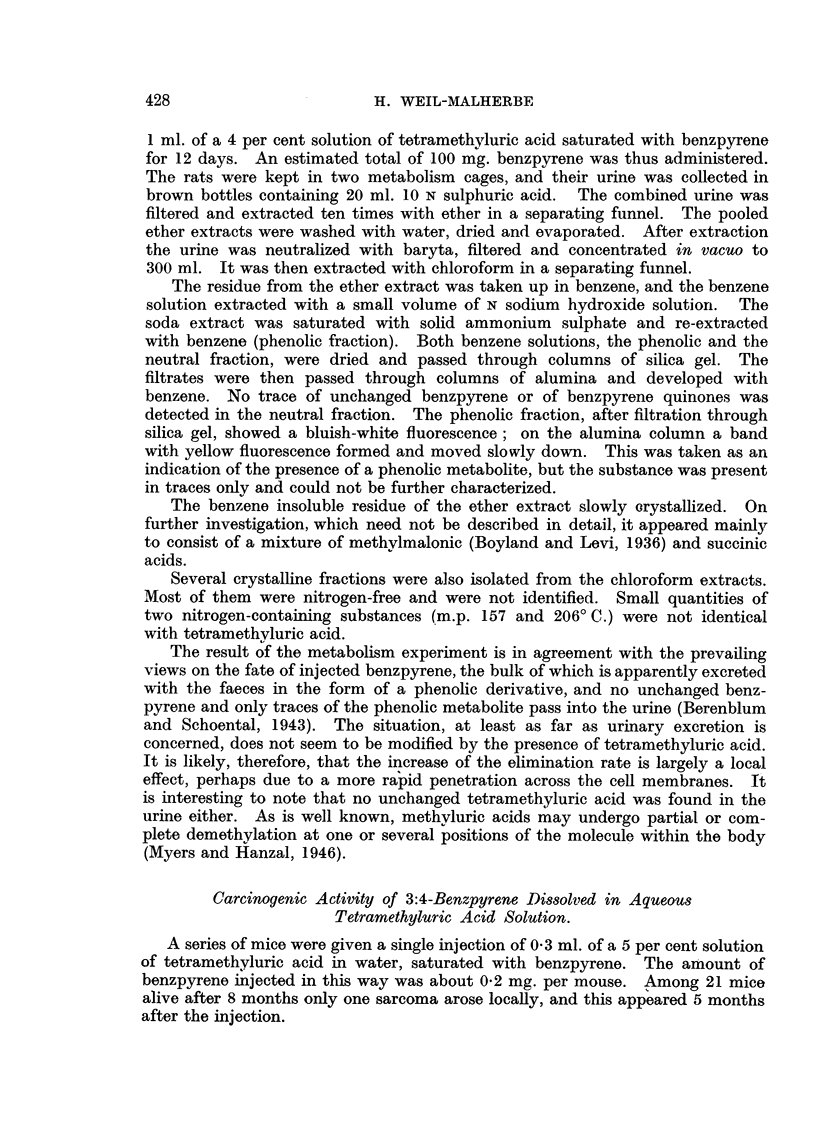

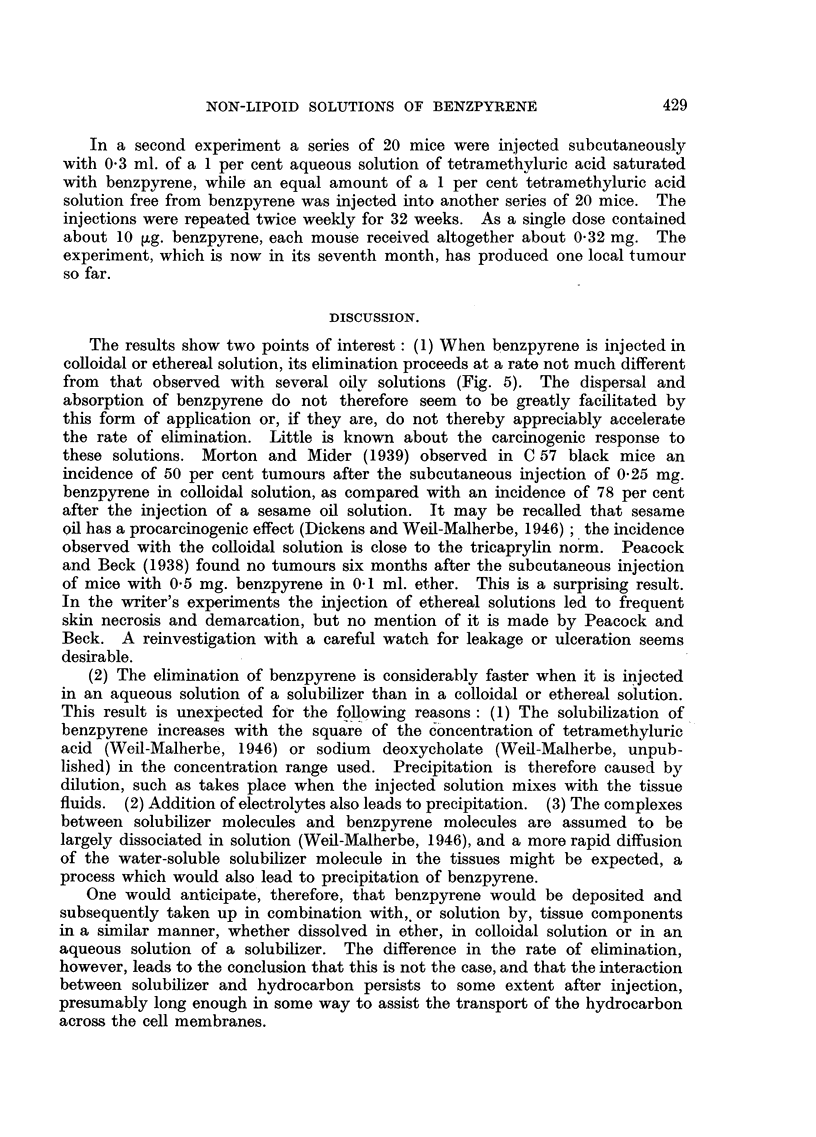

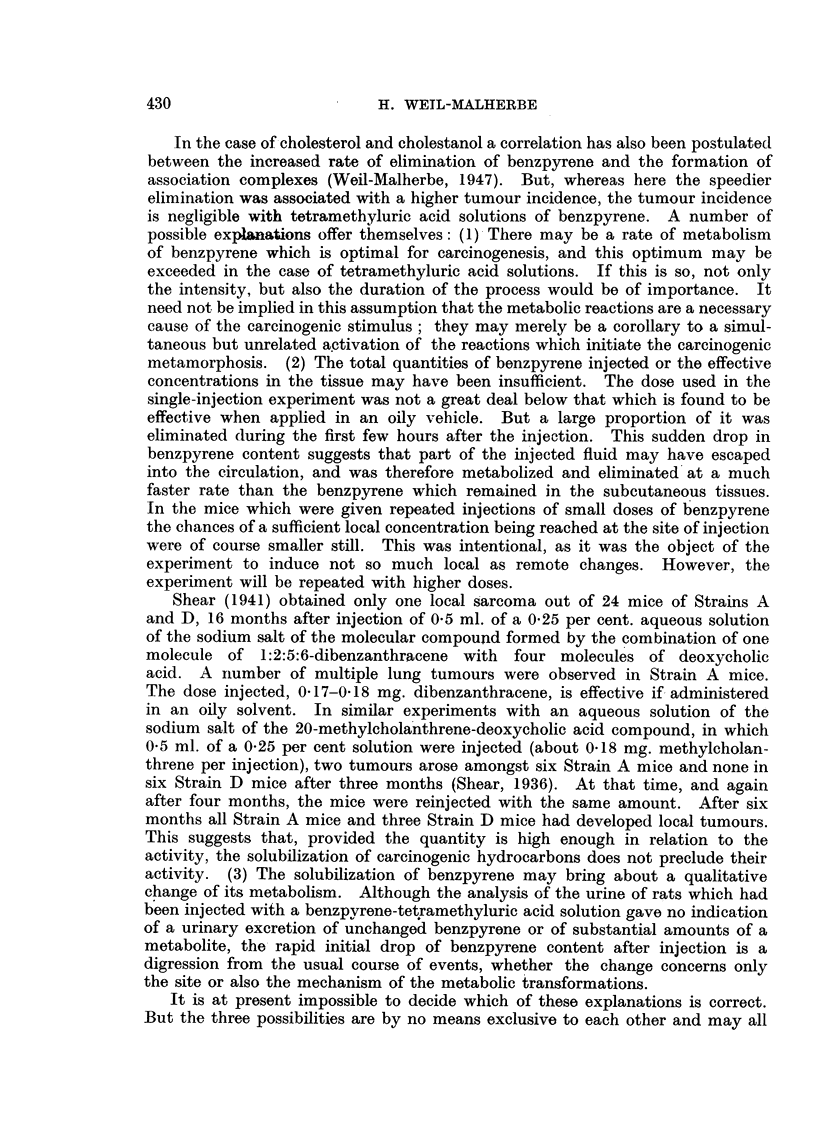

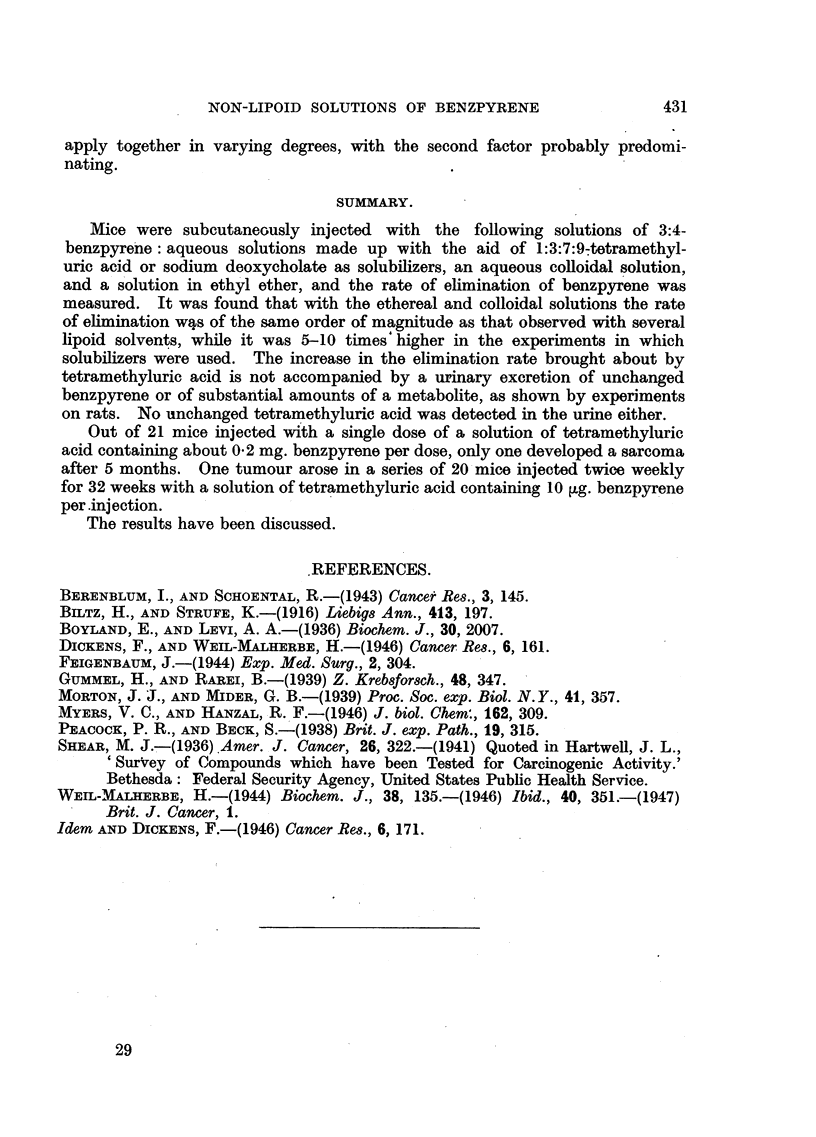


## References

[OCR_00582] Boyland E., Levi A. A. (1936). The isolation of methylmalonic acid from rat urine.. Biochem J.

[OCR_00598] Weil-Malherbe H. (1946). The solubilization of polycyclic aromatic hydrocarbons by purines.. Biochem J.

